# Benefits for African American and white low-income 7–10-year-old children and their parents taught together in a community-based weight management program in the rural southeastern United States

**DOI:** 10.1186/s12889-018-6006-4

**Published:** 2018-09-10

**Authors:** Diane C. Berry, Robert G. McMurray, Todd A. Schwartz, Reuben Adatorwovor

**Affiliations:** 1 The University of North Carolina at Chapel Hill, School of Nursing, Campus Box 7460, Chapel Hill, NC 27599-7460 USA; 2 Department of Exercise and Sport Science, The University of North Carolina at Chapel Hill, Campus Box 8700, Chapel Hill, NC 27599-8700 USA; 3 Department of Biostatistics, Gillings School of Global Public Health and School of Nursing, The University of North Carolina at Chapel Hill, Campus Box 7420, Chapel Hill, NC 27599-7420 USA

**Keywords:** Behavior, Culturally competent interventions, Dietary intake, Exercise, Family intervention

## Abstract

**Background:**

Low-income children and parents are at increased risk for developing overweight and obesity. Therefore, the purpose of this exploratory study was to compare whether African American and white children and parents benefitted equally from a community-based weight management intervention delivered in two rural counties in southeastern North Carolina (N.C.).

**Methods:**

We compared the efficacy of the Family Partners for Health intervention for African American and white children and their parents by testing the three-way interaction of the intervention group according to visit and race.

**Results:**

African American children in the intervention group weighed significantly (*P* = 0.027) less than those in the control group, while white children in the intervention group weighed less than those in the control group, but the difference did not reach statistical significance. African American and white parents in the intervention group weighed less than their respective control groups across all three data collections, but the difference was only significant in the group of white parents (*P* = 0.010) at the completion of the study. At the completion of the study, African American children in the intervention group received significantly (*P* = 0.003) more support for physical activity than African American children in the control group. At both time points, white children in the intervention group were not significantly different from those in the control group. African American parents in the intervention group scored slightly worse in the stress management assessment compared to those in the control group, while white parents in the intervention group showed a significantly (*P* = 0.041) better level of stress management than those in the control group. At the completion of the study, African American parents in the intervention group scored somewhat worse in emotional eating self-efficacy compared to the scores of the African American parents in the control group, while white parents in the intervention group scored significantly (*P* < 0.001) better than those in the control group.

**Conclusions:**

We were successful in affecting some outcomes in both African American and white children and parents using the same intervention.

**Trial registration:**

NCT01378806 Registered June 22, 2011.

## Background

Overweight and obesity continue to increase in ethnic minority children and parents in the United States (U.S.) and globally [1]. Overweight in children is defined as a body mass index (BMI) percentile for age and gender between the 85.0th and 94.9th percentile, and obesity is defined as a BMI percentile at or above the 95.0th percentile [1]. Overweight in adults is defined as a BMI between 25.0 kg/m^2^ and 29.9 kg/m^2^, and obesity is defined as a BMI of 30.0 kg/m^2^ or above [1]. Overweight and obesity in children and adults increases the risk of developing prediabetes, type 2 diabetes, and cardiovascular disease later in life [2, 3]. Currently in the U.S., 38% of children are overweight and 20% are obese, and 69% of adults are overweight and 22% are obese [4]. However, ethnic minority children and parents are disproportionately affected, with 46% of African American children overweight or obese compared to 38% of white children [4]. Additionally, 78% of African American adults and 69% of white adults are overweight or obese [4]. Medical expenditures for obesity range from $147–210 billion dollars per year in the U.S. [5].

Over the past four decades, nutritional intake has changed dramatically for children and adults, resulting in excessive calorie and fat intake [6]. The consumption of sugary drinks and highly processed and fried foods continues to increase, while fruit and vegetable consumption continues to decrease in both children and adults [6]. The U.S. Department of Health and Human Services and the U.S. Department of Agriculture have set forth new dietary guidelines for Americans [7]. These include that caloric intake should be adequate to sustain healthy growth in children and maintain or reduce weight in adults [7]. Dietary recommendations for adults and children include an abundance of fruits, vegetables, whole grains, and lean meat and fish and recommend limiting fast food, sugary beverages, and fried foods [7].

In the U.S., physical activity has decreased over the last four decades in both children and adults, secondary to the increase in sedentary activities [8]. Physical activity guidelines for children include at least 60 min of activity on most days of the week [8]. Current guidelines for adults include 30 to 60 min of physical activity on most days of the week [8]. However, approximately 50% of children and adults do not meet physical activity guidelines in the U.S. [8].

Weight management in adults is aimed at slow and steady weight loss over time [9]. One to two pounds a week is considered to be reasonable and safe [9]. However, children are still developing. Therefore, weight management is aimed at slowing excessive adiposity and weight gain, and as a child grows taller, they will most likely “grow into” their height [9]. Parents influence the physical health and well-being of their children, and they are responsible for responding to their health needs, adhering to necessary treatments, implementing healthy practices, and instituting preventive measures to ensure that their children remain healthy [10]. Parents influence children’s eating habits through availability of particular foods, portion size and mealtime structure [10]. Parents’ physical activity habits and attitudes also have a strong influence on children’s physical activity [10]. Therefore, it is important for both parents and children to receive the same information together so they can improve their nutrition and physical activity behaviors [10].

The majority of interventions for weight management in children and adults focus on nutrition, physical activity, and cognitive behavioral components [9]. Interventions in which children and parents are taught the same weight management program have been found to create partnerships resulting in improved outcomes [10]. To date, there have been few community-based weight management programs that partner African American and white, 7–10-year-old children and a parent together to improve adiposity, weight, health behaviors and eating and exercise self-efficacy [10].

Low-income children and parents from the rural south are at increased risk for developing overweight and obesity. It is imperative to develop and efficacy test interventions focused on nutrition, physical activity and cognitive behavioral components that have potential to be successful in multiple ethnic and racial groups. Therefore, the purpose of this exploratory study was to compare whether African American and white children and parents benefitted equally from a community-based weight management intervention delivered in two rural counties in southeastern North Carolina (N.C.), U.S.

## Methods

### Study design

For this exploratory study, data were analyzed from children and parents from the Family Partners for Health Study, which was a cluster randomized controlled trial [10]. We partnered with eight elementary schools for enrollment and evening delivery of the intervention. A total of 358 child-parent dyads were enrolled over 3½ years from eight rural elementary schools. The intervention included nutrition and exercise education, coping skills training and physical activity for children and parents in the same classroom. The University of North Carolina at Chapel Hill Institutional Review Board (IRB) approved the study. The main study results have been published elsewhere [10].

### Setting

A total of eight rural elementary schools were used as sites for enrollment between August 2007 and April 2010. We met with the superintendents of both school districts and requested that he/she choose four schools in each district that had more than 90% participation in the free lunch program and whose principals would work with us. All eight schools that were approached agreed to participate. There were eight enrollment periods, and 44–45 children and parents were enrolled at two separate schools. The schools were cluster randomized by the study statistician. Additional details on the process have been published elsewhere [11].

### Sample

English-speaking children 7-to-10 years of age and one of their parents were screened. Inclusion criteria for children included an ability to speak, write and read in English; a BMI ≥ 85th percentile; and one parent with a BMI > 25 mg/kg^2^. The child had to live with the parent. Inclusion criteria for the parent included an ability to speak, write and read in English; a BMI > 25 mg/kg^2^; a 7–10-year-old child with a BMI > the 85th percentile; and they must have been living with the child. Exclusion criteria for both the child and parent included if either had a heart murmur, history of sudden death in their family history, were claustrophobic or were participating in another weight management program. Assent and consent were obtained before participation [10].

### Intervention

The intervention children and parents attended class together and received a 60-min nutrition and exercise education and coping skills training intervention and a 45-min physical activity intervention weekly for 12 weeks. The first four classes focused on nutrition and concentrated on understanding calories, protein, carbohydrates and fat, portion control, healthy substitutes, and choosing healthy food when eating out. The fifth class focused on the importance of exercise. Classes six through eleven on coping skills training focused on increasing exercise (cognitive restructuring), improving nutrition and exercise behaviors (social problem solving), motivating each other in a positive manner (assertiveness training), understanding barriers to healthy choices (social problem solving) and working through conflict (conflict resolution). The twelfth and final weekly class was focused on pulling all that they learned together [11]. Then, they received a 60-min nutrition and exercise education and coping skills training intervention and a 45-min physical activity intervention monthly for 9 months. Then, they had 6 months with no contact from the study staff. The intervention group parents and children received a total of 21 classes. The full protocol for the intervention, main results, recruitment and retention has been published elsewhere [10–12].

Therefore, the purpose of this exploratory study was to compare whether African American and white children and their parents benefitted equally from a community-based weight management intervention delivered in two rural counties in southeastern N.C.

### Instruments

All data were collected on intervention and control group parents and children by trained research assistants who were blinded to the study group [10, 11]. The data were available for this exploratory study. Demographic data for the parents and their children were collected from the parents. The following data were collected from both the children and parents: weight, adiposity, health behaviors, and self-efficacy [10, 11]. Data were collected at baseline (0 months), post phase one intensive intervention (3 months), post phase two continued support intervention (12 months), and after 6 months with no contact from the study staff (18 months) [10, 11].

#### Demographic data

Parents provided information on their and their children’s age, date of birth, gender, race, ethnicity, and socioeconomic status. Additionally, information was collected on their and their children’s health status.

#### Body mass index and body mass index percentile

For both children and parents, height was measured twice in street clothes without shoes using a stadiometer that was calibrated at 1/8 cm intervals. Weight was measured twice in light clothing and without shoes on a Tanita WB110A Digital Scale (Tanita, Arlington Heights, IL, USA). An average of the two measurements was used for further analysis. BMI for parents and BMI percentile for children were calculated on a computer.

#### Adiposity

For both children and parents, waist circumference, triceps and subscapular skinfolds were measured three times and averaged. These measures were taken according to the National Health and Nutrition Examination Survey Procedures on the right side of the body [13]. The entire procedure, including inter-rater reliability, has been published elsewhere [10].

#### Health behavior outcomes

The Child Health Behavior Survey [14] was collected from children, and the Adult Health Behavior Survey [14] was collected from parents. Both versions examine trends across time on fast food consumption, beverage intake, and fruit and vegetable intake. The Child and Adolescent Health (CATCH) survey was used to examine health behaviors in the children. The CATCH survey contains 130 items in seven subscales and uses a three-point Likert scale [15]. The alpha coefficients for the subscales ranged from 0.49 to 0.83 [15]. The Health Promoting Lifestyle Profile II (HPLP II) was used to examine health behaviors in the parents [16]. The nutrition, exercise, health responsibility and stress management scales were used [16]. The HPLP II has a total of 48 questions with a choice of four responses, including never, sometimes, often and routinely [16]. The alpha coefficients range from 0.78 to 0.93 for the subscales [16].

#### Self-efficacy outcomes

Eating and exercise self-efficacy was measured in the children and their parents. The CATCH eating and self-efficacy scales were used to collect information on the children’s eating and exercise self-efficacy [15]. The alpha coefficients for eating self-efficacy were 0.73, and exercise self-efficacy was 0.49 [15]. To measure eating self-efficacy in the parents, the Eating Self-Efficacy Scale was used [17]. The Eating Self-Efficacy Scale has 25 questions and two subscales, which include socially acceptable eating and negative affect eating [17]. Parents choose a number between 1, which equates to no difficulty, to 7, which equates to great difficulty [17]. The alpha coefficient for the negative affect subscale was 0.97, and the positive affect scale was 0.93 [17]. Exercise self-efficacy in the parents was measured using Bandura’s Exercise Self-Efficacy Scale [18]. The scale has 18 questions and ranges from 0 to 100 in 10-point ranges, with 0 meaning they do not feel they can do it, 50 meaning that they can moderately do it and 100 meaning that they are certain they can do it [18]. The questions are summed and divided by 18 to calculate a mean score [18]. The alpha coefficient was 0.95 [18].

### Data analysis

The Statistical Analysis Software (SAS, Cary, NC, USA) version 9 was used for analysis.

Using general linear mixed effects modeling, we compared the efficacy of the intervention for African American and white children and their parents via testing of the three-way interaction of intervention group by visit and race. This approach conceptually examined racial heterogeneity of the effect of the intervention across the follow-up visits. These models included a random effect to account for within-cluster correlation, as well as within-person correlation due to repeated measures. These *p*-values were not corrected for multiplicity due to the exploratory nature of this analysis, as well as considering the reduced statistical power for testing the three-way interaction term. Significance was nominally specified at the 0.05 level.

## Results

The baseline demographic characteristics of the intervention and control groups were not significantly different by race for the parents in age, gender, marital status, employment level, education level or income level and for the children in age, gender or education level (Table 1). The aim of this exploratory study was to determine whether African American and white children and parents in the intervention group benefitted equally from the intervention in weight, adiposity, health behaviors and self-efficacy. These exploratory results are presented as estimates of the intervention versus control group mean differences, along with their standard errors. For the purpose of brevity, only response variables with significant three-way intervention group by visit by race interactions are reported.

**Table 1:** Baseline Demographic Characteristics of Intervention and Control Groups by Race

Variable	African American						Whites					
	Total		Intervention		Control		Total		Intervention		Control	
	*N*	%	*N*	%	*N*	%	*N*	%	*N*	%	*N*	%
Parent												
Age	36.2 ± 8.3		36.0 ± 8.2		36.4 ± 8.4		38.4 ± 7.4		38.6 ± 7.5		38.1 ± 7.4	
Body Mass Index (kg/m^2^)	38.6 ± 8.4		36.9 ± 8.3		40.4 ± 8.2		36.1 ± 8.1		35.2 ± 8.0		37.2 ± 8.2	
Gender												
Male	10	4.59	5	4.27	5	4.95	11	10.38	6	10.91	5	9.80
Female	208	95.41	112	95.73	96	95.05	95	89.62	49	89.09	46	90.20
Marital status												
Married	74	33.94	42	35.90	32	31.68	74	69.81	40	72.73	34	66.67
Divorced	38	17.43	18	15.38	20	19.80	24	22.64	10	18.18	14	27.45
Never married	85	38.99	44	37.61	41	40.59	6	5.66	4	7.27	2	3.92
Living with someone	21	9.63	13	11.11	8	7.92	2	1.89	1	1.82	1	1.96
Employment												
Full-time	128	58.72	65	55.56	63	62.38	55	51.89	29	52.73	26	50.98
Part-time	21	9.63	13	11.11	8	7.92	11	10.38	6	10.91	5	9.80
Homemaker	18	8.26	12	10.26	6	5.94	21	19.81	11	20.00	10	19.61
Unemployed	51	23.39	27	23.08	24	23.76	19	17.92	9	16.36	10	19.61
Occupation												
Professional	49	22.48	25	21.37	24	23.76	23	21.70	13	23.64	10	19.61
Technical	169	77.52	92	78.63	77	76.24	83	78.30	42	76.36	41	80.39
Education level												
Less than high school	21	9.63	11	9.40	10	9.90	5	4.72	1	1.82	4	7.84
High School	79	36.24	45	38.46	34	33.66	31	29.25	14	25.45	17	33.33
College Diploma	118	54.13	61	52.14	57	56.44	70	66.04	40	72.73	30	58.82
Income												
< $20,000	76	34.86	43	36.75	33	32.67	30	28.30	15	27.27	15	29.41
$20,000–$39,000	87	39.91	38	32.48	49	48.51	35	33.02	18	32.73	17	33.33
≥ $40,000	26	11.93	19	16.24	7	6.93	33	31.13	17	30.91	16	31.37
Do not wish to respond	29	13.30	17	14.53	12	11.88	8	7.55	5	9.09	3	5.88
Biological parent												
Yes	31	14.22	21	17.95	10	9.90	12	11.32	5	9.09	7	13.73
No	187	85.78	96	82.05	91	90.10	94	88.68	50	90.91	44	86.27
Children												
Age	8.6 ± 1.0		8.7 ± 1.0		8.6 ± 1.0		8.5 ± 0.9		8.6 ± 1.0		8.4 ± 0.8	
Body Mass Index Percentile	96.2 ± 4.7		95.3 ± 5.3		97.2 ± 3.8		95.3 ± 5.6		94.6 ± 5.6		96.0 ± 5.6	
Body Mass Index Z-Score	2.0 ± 0.5		1.9 ± 0.6		2.2 ± 0.5		1.9 ± 0.5		1.8 ± 0.5		2.0 ± 0.5	
Gender												
Male	98	44.95	57	48.72	41	40.59	44	41.51	20	36.36	24	47.06
Female	120	55.05	60	51.28	60	59.41	62	58.49	35	63.64	27	52.94
Education level												
2nd grade	41	18.81	21	17.95	20	19.80	20	18.87	11	20.00	9	17.65
3rd grade	89	40.83	47	40.17	42	41.58	50	47.17	22	40.00	28	54.90
4th grade	88	40.37	49	41.88	39	38.61	36	33.96	22	40.00	14	27.45

In regards to child weight at the end of phase one of data collection (3 months), both African American and, more so, white children in the intervention group demonstrated lower weights than the children in the control group, though the differences were not significant (Table 2). However, at phase two of data collection (12 months), African American children in the intervention group exhibited weights that were significantly lower than the children in the control group, while the white children in the intervention group exhibited a nonsignificant increase compared to the white children in the control group. At the completion of the study (18 months), the weight of the African American children in the intervention group were significantly lower than those of the control group, while the weights of the white children in the intervention group were lower than those of the control group, but the difference did not reach significance.

**Table 2:** African American versus White Intervention and Control Children’s and Parent’s Benefits from the Intervention

Variable	Intervention versus Control Post Phase One (3 Months) Estimate (SE)	Intervention versus Control Post Phase Two (12 Months) Estimate (SE)	Intervention versus Control Completion of Study (18 Months) Estimate (SE)	F (numerator df, denominator df)	*P*
Child Race					
Child Body Mass Index Percentile =				1.17(2, 683)	0.310
African American	−0.595(0.717)	−1.158(0.748)	− 0.555(0.759)		
White	−1.493(1.172)	− 0.263(1.223)	0.124(1.228)		
Child Weight (Kilograms) =				3.65 (2, 683)	0.027
African American	−0.173 (0.546)	−1.516 (0.574)*	− 2.354 (0.585) *		
White	−0.708 (0.888)	0.918 (0.937)	−0.775 (0.943)		
Child Support for Physical Activity from Parents #				5.96 (2, 688)	0.003
African American	0.628 (0.673)	2.077 (0.710)*	1.503 (0.721) *		
White	2.923 (1.112) *	0.180 (1.175)	−0.626 (1.182)		
Parent Race					
Parent Body Mass Index (kg/m^2^) =				3.58 (2, 688)	0.028
African American	−0.483 (0.359)	−0.988 (0.376)	− 0.668 (0.378)		
White	−0.363 (0.513)	−0.974 (0.551)	−2.011 (0.552) *		
Parent Weight (Kilograms) =				4.67 (2, 688)	0.010
African American	−0.829 (0.760)	−1.553 (0.804)	− 0.944 (0.808)		
White	−0.771 (1.113)	−2.365 (1.209)	−5.102 (1.212) *		
Parent Weight Change (Percent) =				5.50 (2, 688)	0.004
African American	−0.876 (0.768)	−1.731 (0.812)*	− 1.077 (0.816)		
White	−0.823 (1.120)	−2.469 (1.216) *	− 5.575 (1.220) *		
Parent Stress Management #				3.20 (2, 695)	0.041
African American	0.140 (0.078)	0.047 (0.082)	−0.010 (0.082)		
White	0.157 (0.109)	0.233 (0.117)*	0.341 (0.117) *		
Parent Negative Affect Eating Self-Efficacy =				7.53 (2, 695)	< 0.001
African American	−0.232 (0.166)	−0.102 (0.175)	0.084 (0.175)		
White	−0.038 (0.254)	−0.516 (0.274)	− 0.977 (0.274) *		

Only significant interactions are reported; *P* values are for the test of interaction between intervention group and race and uncorrected for multiple comparisons; = a negative (−) score is better; # a positive (+) score is better

df = degrees of freedom

*p < .05

In regards to support for physical activity at the completion of phase one of data collection, African American and, more so, white children in the intervention group both received more support for physical activity than their counterparts in the control group, but this was significant only for the white children. However, at phase two of data collection and upon completion of the study, African American children in the intervention group received significantly more support for physical activity than African American children in the control group. At both of those time points, white children in the intervention group were not significantly different from the control group.

African American and white parents’ BMIs and weight changes in kilograms exhibited similar patterns: the intervention group was lower compared to their respective control groups across all three data collections, but only white parents reached significance at the completion of the study. For percent weight change, the same pattern held true, but both African American and white parents at phase two of data collection were significant. However, only white parents reached significance at the completion of the study.

Stress management in African American and white parents in the intervention group was consistently better than African American and white parents in the control group across phase one and phase two of data collection, though only significantly so for the white parents at phase two of data collection. By the completion of the study, African American parents in the intervention group scored slightly worse for stress management compared to the control group, while the white parents in the intervention group showed significantly improved stress management compared to the control group.

Scores for negative affect or emotional eating self-efficacy in African American and white parents in the intervention group were found to be consistently higher than the respective control groups across both the post phase one and post phase two data collections, though none of these comparisons were significant. However, by the completion of the study, African American parents in the intervention group scored somewhat worse in emotional eating self-efficacy compared to African American parents in the control group, while white parents in the intervention group scored significantly better than their control group.

## Discussion

African American and white children are clearly facing an epidemic of overweight and obesity in the United States [1]. There is increased interest in finding interventions that are easy to deliver and scale up quickly and that are applicable to large groups of children and parents to manage and prevent overweight and obesity [19]. Interventions that can serve both African American and white children and parents are needed to improve health outcomes. However, few interventions have been conducted in African American and white low-income, rural children and parents [10, 11]. This study is notable in that it is one of the first studies in which African American and white school age, low-income, rural children from the southern U.S. were taught together with their parents in the same program.

This study demonstrated that African American and white children and their parents benefited relatively equally across the board, though sometimes slightly more or slightly less than the other group, depending on the variable. In regards to weight, African American children in the intervention group exhibited significantly lower weights than the control group at the end of phase two and at the end of the study. It is encouraging that African American children had lost more weight than both the African American control group and the white children in the intervention and control groups. Current statistics confirm that African American children carry a higher burden of overweight and obesity when compared to white children [20]. From 2011 to 2012, approximately 20.5% of African American female children were obese compared to 15.6% of white female children, and 19.9% of African American male children were obese compared to 12.6% of white male children in the 2-to-19-year-old age group [20]. Therefore, an intervention that is beneficial for both African American and white children and their parents, such as that used in the current study, has potential to be utilized in public schools and community centers throughout the state and nationally.

White children initially received more support for physical activity from their parents; however, by phase two and at the completion of the study, African American children received more support for physical activity from their parents. Receiving positive feedback from parents for physical activity endeavors is very important for all children [21]. African American parental role modeling and encouragement to be physically active has been shown to be an important aspect of children’s physical activity levels [22]. The intervention in this current study provided positive messaging for both African American and white children and their parents. During each nutrition and physical activity class, the interventionists encouraged parents and children to discuss their family traditions in food preparation, and together, the group and the interventionists discussed how to improve recipes that had been in the family for many years. Many African American and white parents shared recipes with each other and brought feedback to subsequent classes. Similarly, the interventionists encouraged parents and children to modify different types of physical activity they could work into their everyday lives so it felt comfortable and safe.

In regards to African American and white parents’ BMI change, weight change in kilograms, and weight change in percent, both racial groups were moving in the right direction and appeared to benefit from the intervention; however, the effect was more pronounced by the completion of the study for white parents. These findings are similar to a systematic review conducted by Tussing-Humphreys and colleagues [23] on behavioral lifestyle interventions in African American women in which they found that, overall, African American women had more difficulty than white women in losing weight and maintaining weight loss. Further research is needed to examine these discrete differences more carefully.

The effects of the intervention on parents’ stress management and negative affect or emotional eating self-efficacy were most strongly seen in the post phase two and upon completion of the study for white parents compared to African American parents. Low-income parents many times feel a disproportionate level of stress secondary to employment, housing, transportation, financial hardships, and the inability to meet the basic needs of their families [24]. Diggins and colleagues [25] found an association between perceived stress, contextualized stress and emotional eating among African American women. Further research is needed to more fully understand the association between stress and emotional eating in African American women and how to fine-tune the intervention to meet African American women’s needs better.

### Limitations

The limitations of the study include that the data do not reflect a representative sample of all African American and white children and parents. These children and parents were from the rural southeastern U.S. In addition, African American and white children and parents may have differing cultural traditions regarding food preparation and physical activity. Additionally, we highlight the exploratory nature of these subgroup analyses and emphasize their usefulness in hypothesis-generation, rather than being seen as confirmatory findings. Data were self-reported except for height, weight and adiposity, and potential bias is possible with self-reported data. Children at this age are still increasing in height, weight and, many times, adiposity. Therefore, looking at weight alone instead of height and weight (BMI) may have not have given a clear picture of how the children were growing. The models included in the analysis used a random effect to account for within-cluster correlation, as well as accounting for within-person correlation due to repeated measures. These *p*-values were not corrected for multiplicity due to the exploratory nature of this analysis, as well as considering the reduced statistical power for testing the three-way interaction term. Despite these limitations, the study provides important information for a large group of overweight and obese low-income African American and white children and parents in rural North Carolina. Directions for future research include effectiveness testing in public health departments and scaling up the intervention.

## Conclusions

This study is also notable in that it is one of the first studies in which African American and white school age, low-income, rural children were taught together with their parents in the same program. The study shows that African American and white children and parents respond to the same information and behavioral cues delivered in a weight management program. There were subtle differences; however, overall, the intervention was successful in affecting some weight outcomes in both African American and white children and parents. Children and parents may need a more intensive intervention, strategically placed booster sessions, and longer monitoring to keep the momentum going. The study has implications for future community-based and school-based programs.

## Abbreviations

BMI: Body mass index
Kg: Kilogram
M^2^: Meters squared
U.S.: United States
